# Computational analysis of binding between malarial dihydrofolate reductases and anti-folates

**DOI:** 10.1186/1475-2875-9-65

**Published:** 2010-03-02

**Authors:** Kiattawee Choowongkomon, Sasikrit Theppabutr, Napat Songtawee, Nicholas PJ Day, Nicholas J White, Charles J Woodrow, Mallika Imwong

**Affiliations:** 1 Department of Biochemistry, Faculty of Science, Kasetsart University, Jatuchak, Bangkok 10900, Thailand; 2 Department of Clinical Tropical Medicine, Faculty of Tropical Medicine, Mahidol University, Phayathai, Bangkok 10400, Thailand; 3 Centre for Infection, Division of Clinical Sciences, St George's, University of London, Cranmer Terrace, London, SW17 0RE, UK; 4 Centre for Tropical Medicine, University of Oxford, Churchill Hospital, Oxford, UK

## Abstract

**Background:**

*Plasmodium falciparum *readily develops resistance to the anti-folates pyrimethamine and proguanil via a characteristic set of mutations in the dihydrofolate reductase (PfDHFR) gene that leads to reduced competitive drug binding at the enzyme's active site. Analogous mutations can be found in the DHFR gene in isolates of *Plasmodium vivax *(PvDHFR) although anti-folates have not been widely used for the treatment of this infection. Here the interactions between DHFR inhibitors and modelled structures of the DHFR enzymes of *Plasmodium malariae *(PmDHFR) and *Plasmodium ovale *(PoDHFR) are described, along with an investigation of the effect of recently reported mutations within PmDHFR.

**Methods:**

DHFR models for PmDHFR and PoDHFR were constructed using the solved PfDHFR-TS and PvDHFR structures respectively as templates. The modelled structures were docked with three DHFR inhibitors as ligands and more detailed interactions were explored via simulation of molecular dynamics.

**Results:**

Highly accurate models were obtained containing sets of residues that mediate ligand binding which are highly comparable to those mediating binding in known crystal structures. Within this set, there were differences in the relative contribution of individual residues to inhibitor binding. Modelling of PmDHFR mutant sequences revealed that PmDHFR I170M was associated with a significant reduction in binding energy to all DHFR inhibitors studied, while the other predicted resistance mutations had lesser or no effects on ligand binding.

**Conclusions:**

Binding of DHFR inhibitors to the active sites of all four *Plasmodium *enzymes is broadly similar, being determined by an analogous set of seven residues. PmDHFR mutations found in field isolates influenced inhibitor interactions to a varying extent. In the case of the isolated I170M mutation, the loss of interaction with pyrimethamine suggests that DHFR-inhibitor interactions in *P. malariae *are different to those seen for DHFRs from *P. falciparum *and *P. vivax*.

## Background

**Figure 1:**
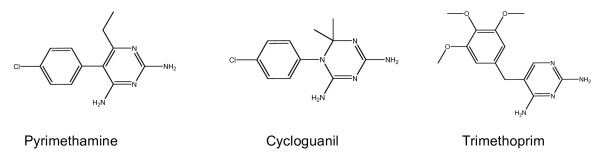
**Two-dimensional representation of anti-folate structures**.

Resistance to anti-malarials is a major cause of morbidity and mortality in tropical countries. Resistance has complicated the treatment of malaria and threatened the control and elimination of the disease. The antifols, a group of drugs that competitively inhibit the folate pathway enzyme dihydrofolate reductase DHFR, and thereby disrupt parasite nucleotide metabolism (Figure 1), were developed in the years following the Second World War. First proguanil (chloroguanide) and then pyrimethamine were deployed extensively, as individual and mass treatments, and as chemoprophylaxis in mass treatment. Resistance developed in both Asia and Africa within a few years of introduction. Combinations with sulphonamides such as sulphadoxine or sulphalene, and sulphones (dapsone) targeting dihydropteroate synthase (DHPS) and synergizing with DHFR inhibition, as well as other classes of drug (e.g. artemisinin derivatives) have retained useful clinical efficacy to varying extents and these drugs remain important treatments in some areas of the world.

Molecular analysis of resistant and sensitive parasite isolates has revealed a characteristic series of mutations in PfDHFR associated with resistance to pyrimethamine and cycloguanil (the active metabolite of proguanil), the two most widely used antifol anti-malarials [1]. These mutations (at residues 16, 50, 51, 59, 108 and 164) have clearly arisen in a particular order, with the primary mutation being S108N in most geographical regions. Molecular and in vitro data from field isolates have been supplemented by heterologous expression studies [2] and the causality of the relationship between genotype and phenotype proven via transfection experiments [3].

Although *Plasmodium vivax *infections are not generally treated with anti-folate therapy, incorrect (i.e. 'clinical') diagnosis and the high frequency of undetected coinfections [4] has inevitably exposed a large number of *P. vivax *parasites to anti-folates, potentially promoting the development of resistance. Anti-folates are efficacious in clearing erythrocytic-stages of *P. vivax *- this was evident in the initial evaluations of proguanil in peninsular Malaya - and subsequent studies confirm efficacy against parasites which are wild-type at the DHFR locus [5]. In areas where anti-folates are used to treat *Plasmodium falciparum*, *P. vivax *dihydrofolate reductase (DHFR) and dihydropteroate synthase (DHPS) mutations have emerged at positions known or predicted to mediate binding of pyrimethamine/cycloguanil [6] and sulphadoxine respectively [7]. PvDHFR displays an array of mutations associated with resistance (at residues 13, 57, 58, 61, 117 and 173) that closely resemble those seen in PfDHFR both in their ordered appearance and in their relative location within the primary amino acid sequence [8]. Heterologous expression studies [9] have shed light on the role of these mutations in mediating resistance.

Like *P. vivax *infections, malaria caused by the two other species which commonly infect humans (*Plasmodium malariae *and *Plasmodium ovale*) is also not conventionally treated with anti-folates. Nevertheless, selection of several PmDHFR mutations corresponding to resistance mutations seen in PfDHFR and PvDHFR has clearly occurred [10]. There is so far no evidence of such mutations in the recently isolated sequence for PoDHFR (Accession no: EU 266601).

The availability of crystal structures for DHFR with (*P. falciparum *[11]) or without (*P. vivax *[6]) the thymidylate synthase (TS) component of the bifunctional DHFR-TS enzyme, complexed with inhibitors as well as the cofactor NADPH, has shed light on the precise interactions between DHFR inhibitors and each protein in both wild-type and certain mutant states. These two species' DHFR proteins possess highly analogous sets of amino acid residues that interact with inhibitors via a series of non-covalent bonds. Comparative study of wild-type and mutant crystal structures has revealed that mutations reduce inhibitor binding either via the loss of critical hydrogen bonds or by altering steric interactions at or near the active site. In addition certain mutations are hypothesized to compensate for existing ones by influencing the catalytic or substrate binding properties of the enzyme. In medicinal terms these studies show the relative vulnerability of the inflexible pyrimethamine structure to mutations at or near the substrate binding site in DHFR molecules of both *Plasmodium *species, compared to other compounds with greater flexibility that retain activity despite such mutations (e.g. WR99210).

This report describes homology-based modelling of the recently obtained PmDHFR and PoDHFR sequences, using the solved crystal structures of PfDHFR (1J31) and PvDHFR (2BL9) respectively as templates. All four wild-type enzymes appear susceptible to pyrimethamine. The effect of three isolated mutations within the modelled *P. malariae *structure was also investigated. These studies provide insights into the binding interactions between pyrimethamine and the DHFR proteins of these two common *Plasmodium *infections of humans.

## Methods

### Homology modelling

Wild-type *P. falciparum *and *P. vivax *DHFR structures (PDB entries 1J3I and 2BL9) were chosen as templates for modelling according to identity scores from standard pairwise alignments; if identity scores were not significantly different similarity scores were used. Models were made via the SWISS-MODEL web site [12]. The Swiss-Model server constructs the coordinates of large gaps (insertions and deletions) in the target-template alignment by using a *de novo *loop modelling technique. Accuracy of each model was determined by the root mean square error for the main chain atoms. The stereochemical quality of the PmDHFR and PoDHFR models was evaluated by the PROCHECK programme using Ramachandran plots [13]. After the models were established, three mutant versions of PmDHFR were also modelled.

### Molecular docking

The drug ligands pyrimethamine, cycloguanil and trimethoprim were drawn in 2D structure and transformed into 3D structure prior to geometric optimization via the SYBYL^® ^7.3 program (Tripos Associates, St. Louis, MO, USA). These ligands were docked to the PmDHFR and PoDHFR models using AutoDock 3.0.5 [14] with rotational bonds for ligands set to be flexible and rotational bonds for the DHFR receptors set to be rigid. All hydrogen atoms and Kollman charges were added into the protein. In brief the steps involved are entry of the files for the modelled protein and ligands into the programme, location of the active site of individual selected ligand, and then allocation of a grid box of 70 × 60 × 60Å (x, y and z, respectively) for the docking region. Docking tasks were then conducted by a genetic algorithm run 50 times for each pair of protein-ligands. The population size was set at 150. The rate of gene mutation and crossover were set at 0.02 and 0.8, respectively. Finally, the most favourable pose was determined from the docking results using scoring functions from AutoDock as well as the FRED program (OpenEye Scientific Software, Inc., Santa Fe, NM, USA.). The poses of ligands with highest scoring functions were visualized by Swiss-Pdb Viewer 4.0.1 [15,16]. A cut-off of 6Å was used to determine significant intermolecular interactions.

### Molecular dynamics simulation

To investigate more detailed interaction between proteins and inhibitors, the best docking models for ligands with PmDHFR and PoDHFR were determined by molecular dynamic stimulation (MD) using the *Simulation *package in Discovery Studio 2.1 (Accelrys Inc., CA) with CHARMm force field. Briefly, the complex was solvated in a 20Å explicit TIP3P water spherical boundary with harmonic restraint using an inhibitor as a centre of mass, and subsequently energy-minimized by the steepest descent and conjugate gradient methods until the system reached 0.001 kcal/mol•Å convergence. The system was then subjected to a 5 ps heating step from 0 to 300 K, a 150 ps equilibration step at 300 K, and finally 150 ps of full MD production at 300 K with NPT ensemble. All simulation steps were run with a time step of 1 fs and coordinates were recorded every 100 fs. The full MD trajectory was considered for analysis. Interaction energy of inhibitors with each individual residue in the binding site was estimated on the *Simulation *package.

## Results

**Figure 2:**
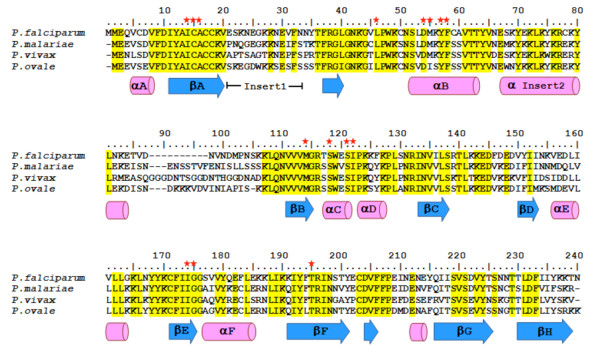
**Multiple alignment of amino acid sequences from four *Plasmodium *DHFRs**. Secondary structure (cylinders and arrows represent helices and strands, respectively) is indicated based on the crystal structure of *P. vivax *DHFR (PDB entry 2BL9). Yellow highlighting represents identical amino acids across the four sequences. Conserved residues in the pyrimethamine binding site of *P. vivax *DHFR are indicated by a red star.

Sequence analysis showed that the four malarial DHFR proteins share high degrees of similarity and identity (Table 1). The alignments used to build models were the specific pairings PfDHFR and PmDHFR (69.9% identity and 83.1% similarity) while for the pair PvDHFR and PoDHFR there was 67.4% identity and 79.1% similarity. These pairings are also consistent with phylogenetic analyses based on other sequences [17,18]. Most amino acids in the core structures of all proteins were highly conserved (Figure 2); at the pyrimethamine binding sites (based on *P. vivax *crystal structure), amino acids are identical among all *Plasmodium *DHFRs. The only two regions that differ among the proteins are the loop inserts after the βA and αI2 domains; neither loop was included in the PvDHFR and PfDHFR crystal structures due to the structural flexibility of these regions.

**Table 1:** Percentage similarity (and identity) for *Plasmodium *DHFR amino acid sequences.

	PfDHFR	PmDHFR	PoDHFR
PvDHFR	77.3 (63.4)	81.1 (70.2)	79.1 (67.4)

PfDHFR		83.1(69.9)	82.6 (64.7)

PmDHFR			86.4 (75.4)

**Figure 3:**
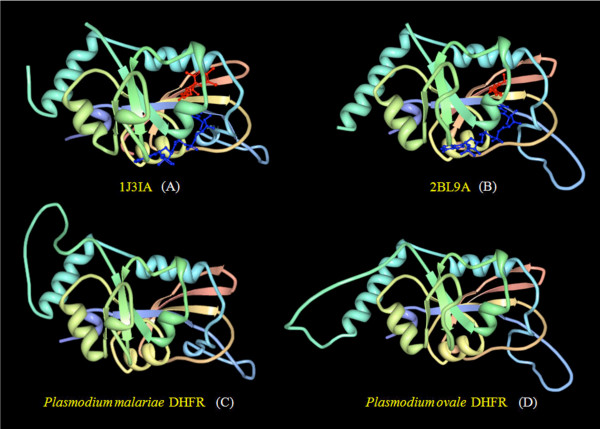
**Three-dimensional structures of *Plasmodium *DHFRs: (A) solved crystal structure of *P. falciparum *DHFR complexed with WR99210 (red ball and stick) and NADPH (blue ball and stick); (B) solved crystal structure of *P. vivax *DHFR complexed with pyrimethamine (red ball and stick) and NADPH (blue ball and stick); (C) homology model of *P. malariae *DHFR, and (D) homology model of *P. ovale *DHFR**.

Overall folding of the homology modelled PmDHFR and PoDHFR structures was similar to that of the crystal structures of PfDHFR and PvDHFR (Figure 3). Root mean square (RMS) deviations of backbone atoms (excluding the missing loop regions) between modelled DHFRs and template structures were significantly low at 0.15Å (PoDHFR vs. PvDHFR) and 0.73Å (PmDHFR vs. PfDHFR), indicating models of high accuracy consistent with the percentage identity of the templates. Ramachandran plot analysis for the PmDHFR model revealed 88.0% of residues were in core (favoured) regions, 11.1% in allowed regions, 0.9% in generously allowed regions and 0.0% in disallowed regions. For PoDHFR the figures were 86% in favoured regions, 11.6% in allowed regions, 1.9% in generously allowed regions and 0.5% in disallowed regions (see Additional File 1). These data indicate satisfactory stereochemical quality.

**Figure 4:**
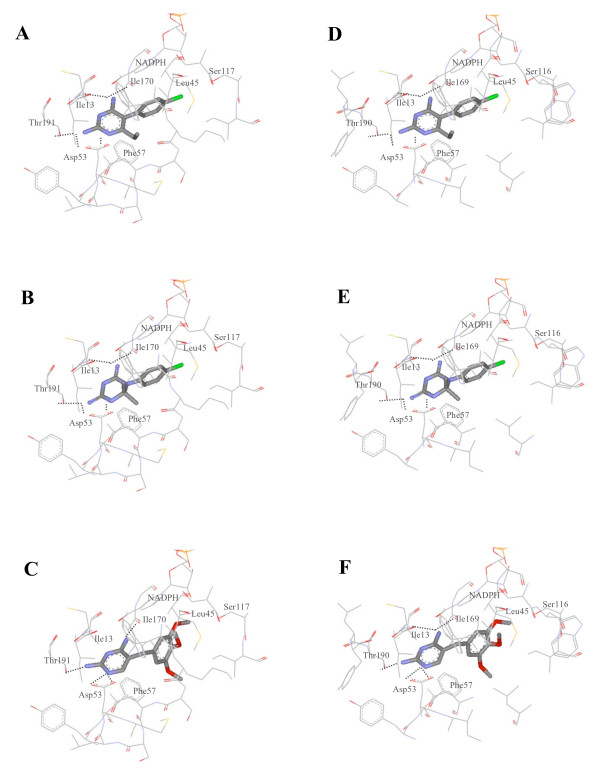
**Docking of compounds into modelled DHFRs**. Pyrimethamine (A), cycloguanil (B) and trimethoprim (C) were docked into the PmDHFR binding site and pyrimethamine (D), cycloguanil (E) and trimethoprim (F) into the PoDHFR binding site.

In order to investigate the binding of pyrimethamine to the modelled PmDHFR and PoDHFR structures, molecular docking simulations were performed using the Autodock 3.0.5 program using the known co-crystal complex of *P. vivax *DHFR bound to pyrimethamine (2BL9.pdb) [6] as calibrator to validate docking parameters. Computationally derived protein/inhibitor complexes of both PmDHFR and PoDHFR were achieved based on the highest docking energy and closest RMS deviations from the known co-crystal complex; this highest docking energy conformation overlaid very well with the experimental co-crystal complex (RMS deviation 0.76). In the case of cycloguanil and trimethoprim, although there is no co-crystal structure with inhibitor, their similarity in core structure to pyrimethamine can be used to select the computational complexes from the docking results. Docking conformations of ligands occurred at the same site for all three ligands (Figure 4). Furthermore, an identical set of seven amino acids was seen to mediate inhibitor binding in every case. Hydrogen bonds were shown to be present between several residues and the inhibitor structure.

These docking results showed that each inhibitor bound to both PmDHFR and PoDHFR in a similar orientation and binding energy to that seen for Pf and PvDHFR model sequence templates. Docking scoring functions for the four species' DHFR were distributed closely from -10.50 to -11.04 kcal/mol in the case of pyrimethamine and from -10.54 to -10.88, in the case of cycloguanil (Table 2). Experimental determinations of inhibition constants (K_i_) of *P. vivax *and *P. falciparum *against pyrimethamine and cycloguanil were also within a narrow range [19,20]. The docking energy of trimethoprim to DHFR showed greater variability among the four different species.

**Table 2:** Final docked energies for DHFR inhibitors.

DHFR sequence	Pyrimethamine	Cycloguanil	Trimethoprim
WT *P. falciparum*	-11.04	-10.88	-11.32
WT *P. vivax*	-10.60	-10.63	-10.87
WT *P. ovale*	-10.50	-10.54	-10.52
WT *P. malariae*	-10.60	-10.64	-10.93
Mutant *P. malariae*			
N50K	-10.88 (-0.28)	-10.40 (+0.24)	-10.76 (+0.17)
S114N	-10.93 (-0.33)	-10.21 (+0.43)	-10.38 (+0.55)
I170M	-8.62 (+1.98)	-7.87 (+2.77)	-9.83 (+1.10)

Results are based on the Autodock 3.0.5 program; values are kcal/mol. For PmDHFR mutants, relative binding energies (ΔΔG) are shown in brackets (reduction in binding produces a positive ΔΔG and enhanced binding produces a negative ΔΔG).

The interaction energy of DHFR inhibitors with individual amino acids in the active side of each DHFR was calculated by the Discovery Studio programme. Residues Ile14/13/13/13 (Pf/Pm/Pv/Po residue number), Leu46/45/45/45, Asp54/53/53/53, Phe58/57/57/57, Ser111/117/120/116, Ile164/170/173/169, and Thr185/191/194/190 were seen to have significant interactions with all three inhibitors important for binding (see Additional File 2). Although all seven residues were found in the binding site, detailed individual interactions for the seven residues with each inhibitor showed distinct properties across DHFRs. For example the Ser111/117/120/116-pyrimethamine interaction appeared strongest for PmDHFR, intermediate for PfDHFR and weak for PvDHFR. This interaction is thought to involve a hydrogen bond between the serine residue and the phenyl chloride of pyrimethamine. In contrast, the Phe58/57/57/57 interaction (thought to be primarily steric in nature) and the Thr185/191/194/190-pyrimethamine interaction appeared relatively weak for PmDHFR compared to that seen in the two known structures PfDHFR and PvDHFR. For PmDHFR, residues Ile13, Leu45, Asp53, Ser117 and I170 appear to play important roles in binding with pyrimethamine.

**Figure 5:**
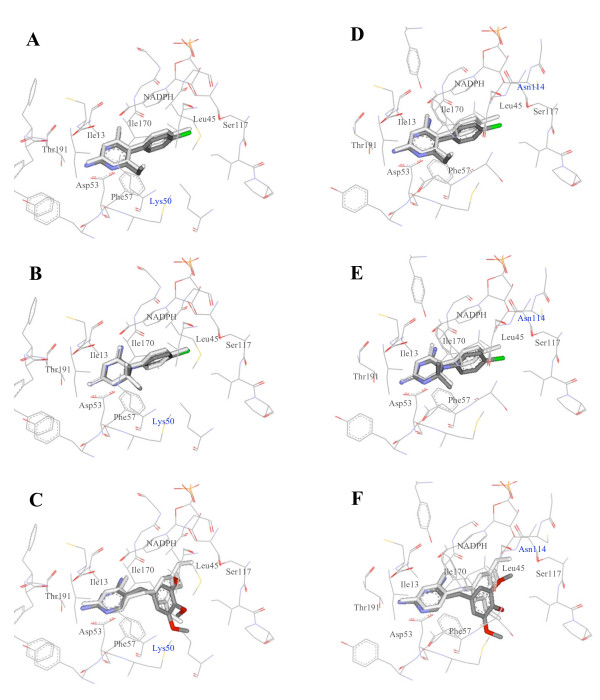
**Effect of PmDHFR N50K and S114N mutations on docking of DHFR inhibitors**. Pyrimethamine (A), cycloguanil (B) and trimethoprim (C) were docked into the PmDHFR-N50K binding site; pyrimethamine (D), cycloguanil (E) and trimethoprim (F) into the PmDHFR-S114N binding site. In each case the ligand in white represents the position when docked to the wild-type protein; the grey/coloured ligand represents docking to the mutant protein.

**Figure 6:**
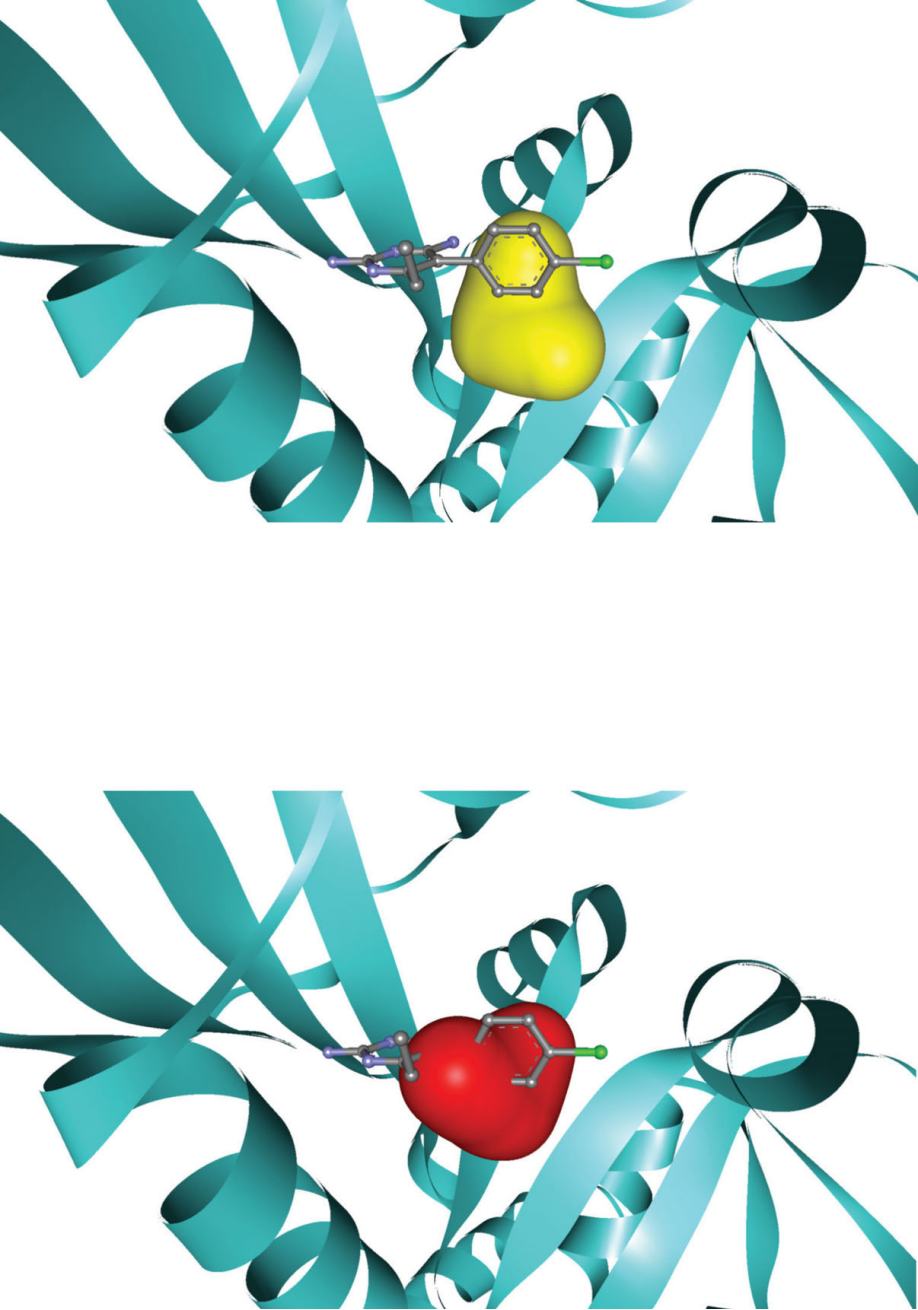
**Effect of PmDHFR I170M mutation on pyrimethamine binding**. A space-filling model is shown for the residue at 170 (in wild-type enzyme isoleucine, shown in yellow and in I170M methionine shown in red).

Sequencing of *P. malariae *isolates has revealed the presence of three isolated mutations in PmDHFR that correlate with those seen in PfDHFR and/or PvDHFR [10]. PmDHFR S114N/G corresponds to S108N and S117N/T in PfDHFR and PvDHFR respectively, mutations, which appear first in field isolates of these species. PmDHFR I170M corresponds to I164L and I173L in PfDHFR and PvDHFR respectively. PfDHFR I164L is associated with high-level resistance to pyrimethamine in *P. falciparum*, but is rarely observed in isolation in this species. PmDHFR N50K corresponds to N51I in PfDHFR but has no known equivalent in PvDHFR. Modelling of these three single mutant proteins revealed an arrangement of side-chains within the ligand-binding site that was comparable to wild-type (Figure 5), except in the case of I170M where the new methionine side-chain caused steric hindrance thereby interfering with inhibitor binding (Figure 6). Calculation of docking energies for the three inhibitors with each mutant revealed that I170M showed significantly reduced docking energy compared to wild-type PmDHFR for all inhibitors with ΔΔG 1.98 kcal/mol for pyrimethamine, 2.77 kcal/mol for cycloguanil and 1.10 kcal/mol for trimethoprim (Table 2). The other mutations had milder effects. PmDHFR S114N appeared to bind cycloguanil less well than wild-type, but pyrimethamine slightly more strongly. N50K appeared to have the least effect of all on inhibitor binding of the three mutations studied.

## Discussion

The emergence of resistance to an anti-malarial drug in a parasite population via a mutation at a specific locus depends on several forces including the fitness advantage that the mutation provides when the parasite encounters drug, and the fitness disadvantage that the mutation confers with regard to normal enzymatic functions. For example, in the case of *P. falciparum *DHFR, mutation S108N has emerged independently on many separate occasions, providing a reduction in pyrimethamine binding to PfDHFR by approximately 10-fold without major adverse effects on the enzymatic properties of PfDHFR [21,22] Conversely, the PfDHFR I164L mutation, the allele most associated with highest levels of pyrimethamine resistance, has appeared in a much more restricted manner, and almost exclusively on a background of several other mutations (including S108N).

For the non-falciparum human malaria species, particularly *P. malariae *and *P. ovale*, these events are much less clear. Despite the fact that patients with these infections are not routinely treated with anti-folates such as pyrimethamine, sequencing of PmDHFR field isolates has shown three mutations, each occurring in isolation, in analogous positions to those seen in PfDHFR and PvDHFR. The suggestion that anti-folates do exert pressure on *P. malariae *and *P. ovale *is supported by the modelling data, which indicate that the binding mode of pyrimethamine, cycloguanil and trimethoprim to all four DHFRs is similar in several respects. The three inhibitors bound to the protein in the same orientation, overall affinity was comparable across species (and ligands), and an identical set of seven residues was present at the pyrimethamine binding site. However, within this overall framework, it was possible to discern differing contributions of individual residues to the binding of ligands across species, in the same manner as noted for the two solved crystal structures of PfDHFR and PvDHFR [6].

The interactions between PmDHFR and anti-folates were explored by modelling the effect of the three putative drug resistance mutations that have been observed in the field for PmDHFR. PmDHFR I170M was associated with substantial losses in docking energy for all three DHFR inhibitors with evidence of steric hindrance by the methionine side chain. It is interesting to note that the homologous replacement is rarely found in isolation in nature for either *P. falciparum *or *P. vivax*. This is likely to reflect the relative enzymatic activity and susbstrate affinity of each mutant protein in isolation. For example, PfDHFR I164L possesses grossly reduced enzymatic activity compared to wild-type PfDHFR when expressed in *E. coli *[23]. This suggests that there are differences between species in terms of the ability of the DHFR enzyme to tolerate mutation at this position; presumably PmDHFR is more tolerant in this regard. Further studies involving heterologous expression of the PmDHFR mutants studied here would be useful in addressing this issue.

The other positions where mutations have been encountered in PmDHFR do not coincide with residues predicted to mediate inhibitor binding by this protein, as seen in the PfDHFR and PvDHFR crystal structures [6]. These mutations act more indirectly on the structure of the binding site; in the case of PvDHFR the S117N mutation causes the subsequent series of residues (118-125) to change orientation with consequent loss of the hydrogen bond between S120, three residues beyond the mutation, and pyrimethamine [6]; PfDHFR S108N shows less displacement of pyrimethamine in the active site than is the case for *P. vivax *S117N [24]. In the model of wild-type PmDHFR, we noted that the electrostatic interaction between pyrimethamine and S117 (three residues beyond the S114N mutation) was stronger than for equivalent residues in Pf and PvDHFR; hence binding may be more resistant to the indirect effect of mutation at S114 than the PfDHFR and PvDHFR cases.

Although the effects were small, PmDHFR S114N resulted in weaker cycloguanil and stronger pyrimethamine binding compared to wild-type enzyme. This somewhat surprising finding may reflect that proguanil (the prodrug of cycloguanil) may have contributed much of the anti-folate selection in Southeast Asia. Ligand-specific alterations in affinity have previously been reported for PfDHFR [25,26]. PmDHFR N50K appeared to have minimal effect on inhibitor interactions. Accurate prediction of these more indirect effects on ligand binding within a molecular model is technically more challenging than for direct effects such as that seen with I170M.

An additional factor that needs to be taken into account when linking field mutations to molecular studies is the contribution of each target enzyme to the pathway under consideration. The finding that an earlier enzyme in the *P. falciparum *folate pathway (GCH1) is amplified in association with PfDHFR mutations known to be directly involved in resistance to DHFR inhibitors illustrates this concept [27]. Quantitative differences in the control of these pathways are also likely to influence the order in which resistance mutations appear in the DHFR enzymes of various species.

## Conclusions

Molecular modelling of the DHFR enzymes of *P. malariae *and *P. ovale *using solved crystal structures as templates led to highly accurate models and confirmed the preservation of enzyme architecture across species. Residues mediating binding to DHFR inhibitors including pyrimethamine corresponded to equivalent residues for *P. falciparum *and *P. vivax*. In the case of *P. malariae*, introduction of the single I170M mutation observed in field isolates led to loss of predicted docking energy with all 3 DHFR inhibitors; however other mutations had smaller effects. The interaction between PmDHFR and inhibitors appears to possess distinct properties compared to empirically determined interactions between inhibitors and DHFRs of *P. falciparum *and *P. vivax*.

## Competing interests

The authors declare that they have no competing interests.

## Authors' contributions

KC, NJW, NPJD, CJW and MI were involved in the conception and design of the study. KC, ST, and NS managed experimental procedure and performed computer work. KC, ST, NS, NJW, NPJD, CJW and MI participated in the statistical analysis. CJW, NJW, NPJD, KC and MI drafted and critically revised the manuscript. All authors read and approved the manuscript.

## Supplementary Material

Additional file 1 **Ramachandran plots of modelled structures of (A) PmDHFR and (B) PoDHFR**. The data provided represent an index of model quality. Click here for file

Additional file 2 **List of *Plasmodium *DHFR residues and binding energies (kcal/mol) for interaction with pyrimethamine, cycloguanil and trimethoprim**. The data provided represent a detailed description of the interactions between conserved residues and three inhibitory ligands. Click here for file
