# Supplementary material for: Computational analysis of binding between malarial dihydrofolate reductases and anti-folates
Source: Malar J. 2010 Mar 2;9:65. doi: 10.1186/1475-2875-9-65 (PMC2838911; doi:10.1186/1475-2875-9-65)
Supplement: Additional file 1 — Ramachandran plots of modelled structures of (A) PmDHFR and (B) PoDHFR. The data provided represent an index of model quality. [file 1475-2875-9-65-S1.PDF]

Additional File 1

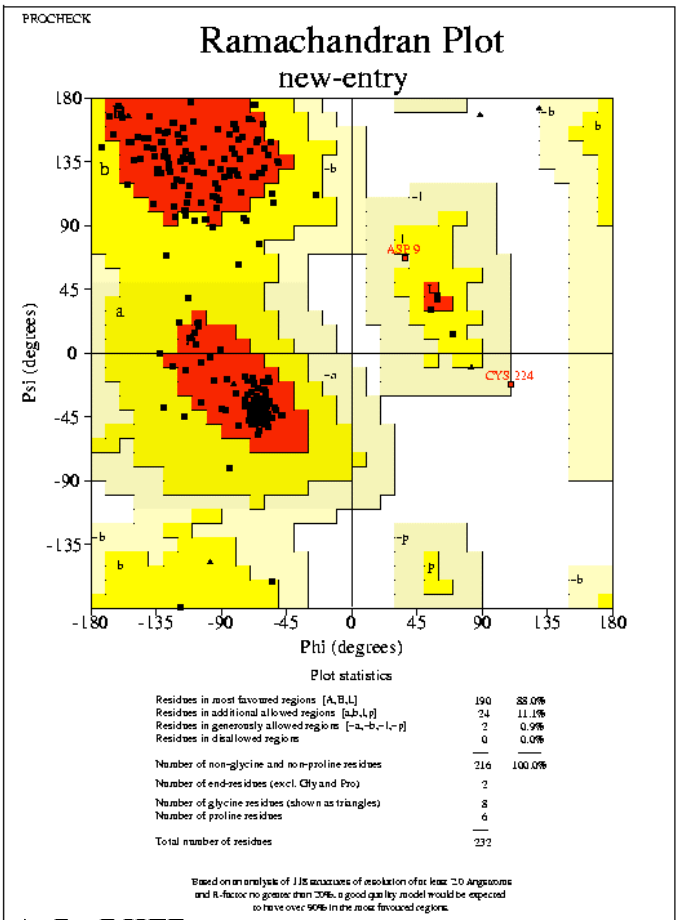

A. PmDHFR

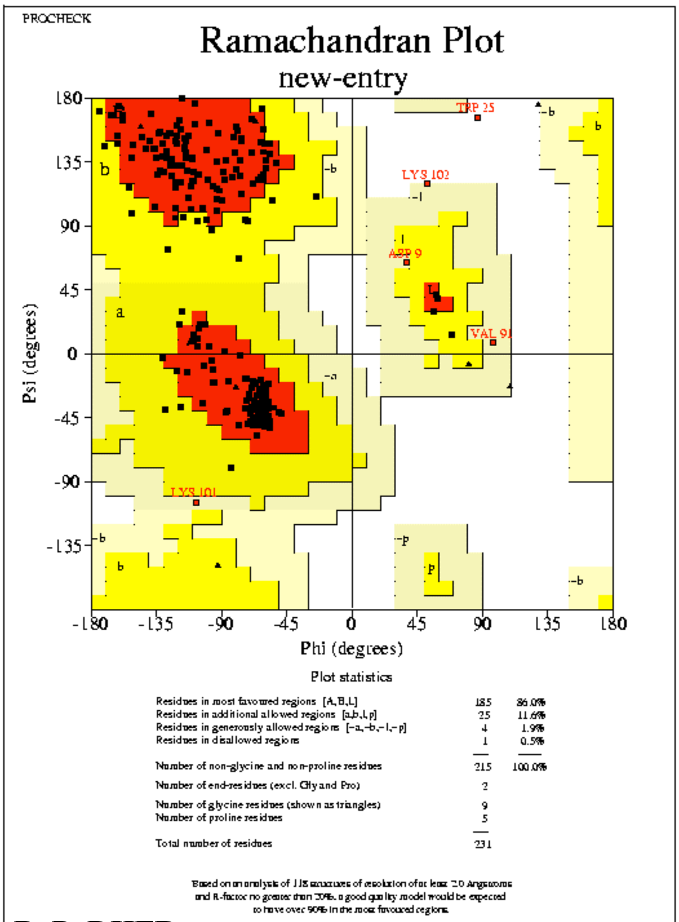

B. PoDHFR
