# Supplementary material for: Computational analysis of binding between malarial dihydrofolate reductases and anti-folates
Source: Malar J. 2010 Mar 2;9:65. doi: 10.1186/1475-2875-9-65 (PMC2838911; doi:10.1186/1475-2875-9-65)
Supplement: Additional file 2 — List of Plasmodium DHFR residues and binding energies (kcal/mol) for interaction with pyrimethamine, cycloguanil and trimethoprim. The data provided represent a detailed description of the interactions between conserved residues and three inhibitory ligands. [file 1475-2875-9-65-S2.PDF]

Additional File 2

	Pyrimethamine			Cycloguanil			Trimethoprim		
P. falciparum	E_{elec}	E_{vdW}	E_{total}	E_{elec}	E_{vdW}	E_{total}	E_{elec}	E_{vdW}	E_{total}
Ile14	-4.45455	-2.85416	-7.30871	-23.94803	1.25755	-22.69048	-4.28407	-0.83837	-5.12244
Leu46	-4.03054	-2.70673	-6.73727	-4.64647	-1.30639	-5.95286	-6.59054	-1.32306	-7.91360
Asp54	-4.79272	-0.48853	-5.28125	-52.78243	1.00131	-51.78113	6.23699	-1.58328	4.65371
Phe58	-0.99061	-5.16104	-6.15165	-0.25851	-3.59562	-3.85412	-3.62644	1.93684	-1.68960
Ser111	-3.02989	-0.02507	-3.05496	-4.25826	-0.89621	-5.15447	-10.52985	-1.83654	-12.36639
Ile164	-2.23773	-3.19442	-5.43215	3.24556	-2.69400	0.55156	-2.12684	-4.91167	-7.03850
Thr185	-3.62890	-0.49249	-4.12139	-4.15436	-0.53773	-4.69208	-2.18125	-0.55655	-2.73780
P. malariae	E_{elec}	E_{vdW}	E_{total}	E_{elec}	E_{vdW}	E_{total}	E_{elec}	E_{vdW}	E_{total}
Ile13	-4.47881	-2.00368	-6.48249	-9.16084	-2.48195	-11.64279	-4.28032	-1.16311	-5.44343
Leu45	-5.07646	-1.85806	-6.93453	-2.26843	-1.51467	-3.78310	-3.97108	-1.18766	-5.15873
Asp53	-3.30848	-2.04229	-5.35077	-43.01132	-1.76586	-44.77718	3.39352	-1.60311	1.79041
Phe57	0.58423	-2.01388	-1.42965	-1.54897	-2.81974	-4.36870	-1.94700	1.56776	-0.37924
Ser117	-5.94840	-0.33789	-6.28630	3.57897	-0.50258	3.07639	-3.31147	-1.01561	-4.32708
Ile170	-3.07075	0.00460	-3.06615	-17.50964	-2.96657	-20.47621	-1.52147	-4.14913	-5.67060
Thr191	2.22904	-0.64117	1.58788	-12.62370	-0.14263	-12.76633	0.52113	-0.92491	-0.40378
P. vivax	E_{elec}	E_{vdW}	E_{total}	E_{elec}	E_{vdW}	E_{total}	E_{elec}	E_{vdW}	E_{total}
Ile13	-5.03716	-0.80234	-5.83950	-30.17255	2.63105	-27.54150	-1.83770	0.84557	-0.99212
Leu45	-0.40744	-1.89968	-2.30712	2.86963	-2.22746	0.64217	-2.84961	-2.60049	-5.45010
Asp53	-4.20510	-1.45995	-5.66505	-55.40000	-1.92449	-57.32449	7.13827	-1.54616	5.59211
Phe57	-1.27598	-5.13095	-6.40693	-0.94481	-3.40577	-4.35058	-5.26190	-3.99911	-9.26101
Ser120	1.95007	-0.22796	1.72211	-4.00818	-0.09603	-4.10421	-4.63495	-0.91358	-5.54853
Ile173	-0.41889	-1.92204	-2.34094	-12.62274	-3.70177	-16.32451	3.44374	-5.50601	-2.06227
Thr194	-4.25289	-0.33762	-4.59052	-4.33977	-0.28285	-4.62262	1.32465	-1.00197	0.32268
P. ovale	E_{elec}	E_{vdW}	E_{total}	E_{elec}	E_{vdW}	E_{total}	E_{elec}	E_{vdW}	E_{total}
Ile13	-3.00702	-2.07637	-5.08339	-15.76425	-3.47074	-19.23499	-4.07571	-1.53574	-5.61145
Leu45	-2.66370	-2.17679	-4.84049	-1.66595	-0.74299	-2.40894	-3.34519	-2.71469	-6.05988
Asp53	-5.38799	-2.65242	-8.04040	-51.30431	-1.90592	-53.21023	0.84022	-0.61648	0.22374
Phe57	-1.06116	-2.79946	-3.86061	0.65365	-4.61498	-3.96133	-4.14499	-1.81602	-5.96101
Ser116	2.22857	-0.24835	1.98022	4.15982	-0.32659	3.83323	-5.22457	-0.57937	-5.80394
Ile169	-1.92536	-0.30377	-2.22914	-22.45711	-2.32643	-24.78354	-0.69062	-5.74079	-6.43141
Thr190	-3.58657	-0.38928	-3.97584	-11.74111	-0.38139	-12.12250	2.13207	-1.19620	0.93587
